# Supplementary material for: Streptomyces sp. AN090126 as a Biocontrol Agent against Bacterial and Fungal Plant Diseases
Source: Microorganisms. 2022 Apr 8;10(4):791. doi: 10.3390/microorganisms10040791 (PMC9025191; doi:10.3390/microorganisms10040791)

Supplementary Materials

Materials and Methods

In vivo assay

Plant material

Tomato ('Seokwnag'; Farm Hannong Co., Ltd, Seoul, South Korea), Kimchi cabbage ('Chunkwang'; Sakada Korea, Seoul, South Korea), and red pepper ('Josaengsintopgochu'; Nongwoobio Co., Ltd., Suwon, South Korea) were sown in vinyl pots (diameter, 6.0 cm) containing nursery soil and maintained in an incubation room with 12 h of daylight per day. The plants were transplanted into plastic pots (diameter, 7.5 cm) 24 h before treatment. Creeping bentgrass was grown in a plastic pot (diameter, 7.5 cm) at 25 °C for 4 weeks, and the potted seedlings were cut at a height of 2 cm from the mouth of the pots 1 day before inoculation.

Tomato bacterial wilt

To tomato plants at the four-leaf stage, the fermentation broth of *Streptomyces* sp. AN090126 was applied using soil drenching. The plant was treated with 20 ml each of the fermentation broth per pot at 24 h before inoculation, and then 20 ml of Rs cell suspension (10^8 CFU/ml, 10 mM $MgCl_2$) was inoculated by soil drenching. The inoculated plants were incubated in the dark for 24 h and then transferred to an incubation room at 30 °C with 75% relative humidity (RH) and 12 h of daylight per day for 10 days. The disease severity was graded on the following scale: 0 = no leaf symptoms; 1 = one leaf wilted; 2 = two or three leaves wilted; 3 = more than four leaves wilted; and 4 = plant death.

Red pepper bacterial leaf spot

The disease control efficacy of the fermentation broth of AN090126 against red pepper leaf spot disease was tested in red pepper plants at the six-leaf stage. Briefly, 5 ml aliquots of each sample were applied to the plants by foliar spray, and then the treated plants were inoculated with 5 ml of Xe cell suspension (10^7 CFU/ml) using the same method 24 h after the treatment. The inoculated plants were incubated at 25 °C with 100% RH and 12 h of daylight per day for 16 days. The disease index was recorded using the following scale: 0 = no symptoms; 1 = symptomless; 2 = a few necrotic spots on a few leaflets; 3 = a few necrotic spots on many leaflets; 4 = many spots with coalescence on a few leaflets; 5 = many spots with coalescence on many leaflets; 6 = severe disease and leaf defoliation; and 7 = plant death.

Creeping bentgrass dollar spot disease

Therefore, the biocontrol efficacy of the fermentation broth was evaluated against dollar spot in creeping bentgrass caused by Sh. Briefly, 4-week-old plants were treated with 20 ml of each sample by soil drenching, and then inoculated with 8 ml of the fungal mycelial suspension (1%) using the same method 24 h after the treatment. The inoculated plants were incubated in a growth chamber at 25 °C with 100% RH and 12 h of daylight per day for 7 days. The disease severity was calculated as the percentage of diseased shoot area.

Kimchi cabbage bacterial soft rot

Briefly, 5-week-old plants were treated with 20 ml of each sample per plant via soil drenching. After 24 h, the treated plants were inoculated with 20 ml of Sr-Pcc suspension (10^7 CFU/ml, 10 mM $MgCl_2$) using the same method. The plants were incubated in the dark at 30 °C with 100% RH for 48 h and then transferred to an incubation room at 30 °C with 12 h of daylight per day for 8 days. The disease severity was assessed using the following scale:

0 = no symptoms; 1 = one or two pencil-line streaks; 2 = more than two pencil-line streaks; 3 = leaf chlorosis or bleaching; 4 = leaf necrosis; and 5 = plant death.

Calculation of the control value

The *in vivo* experiment was repeated two times with three replicates per treatment, and the values were converted to a control percentage (± standard deviation) compared to negative controls using the following equation:

For bacterial disease assays,

Control value (%) = [(DS of untreated plant – DS of treated plant) ÷ DS of untreated plant] × 100% (1)

where DS = disease severity

For fungal disease assay

Control value (%) = [(A – B) ÷ A] × 100% (2)

where A is the infected shoot area (%) in the untreated controls and B is the infected shoot area (%) in the treated plants.

Table S1. Morphological characterization and physicochemical properties of *Streptomyces* sp. AN090126.

**Morphological characterization**	
Color of the substrate mycelium	Greenish gray
Color of the aerial mycelium	Greenish beige
Spore chain arrangement	Long and fragment
Spore surface	Rough
Pigment production	Beige brown
**Enzymatic activity**	
Amylase	-
Cellulase	+
Lipase	+
Protease	+
**Physiological properties**	
0% NaCl	++
1% NaCl	++
3% NaCl	++
7% NaCl	+
10 °C	-
28 °C	+++

45 °C	+++
pH 4.0	+++
pH 7.0	+++
pH 10.0	++

+++, very good; ++, good; + weak; - unresponsiveness

Table S2. Organic acids produced by *Streptomyces* sp. AN090126.

Peak name	Retention time, min	Peak area, %
Oxalic acid	5.940	32.16 ± 0.14[a]
Citric acid	6.552	6.64 ± 0.12[d]
Unknown	6.854	6.60 ± 0.10[d]
Unknown	7.247	5.83 ± 0.06[e]
Tartaric acid	7.839	7.10 ± 0.10[c]
Malic acid	8.215	4.57 ± 0.10[g]
Unknown	8.961	5.63 ± 0.06[f]
Malonic acid	9.472	14.14 ± 0.05[b]
Unknown	10.207	7.05 ± 0.13[c]
Succinic acid	11.001	5.55 ± 0.22[f]
Lactic acid	12.060	1.93 ± 0.03[h]
Unknown	12.747	1.32 ± 0.03[i]
Fumaric acid	13.433	0.21 ± 0.02[m]
Acetic acid	14.940	0.42 ± 0.03[k]
Unknown	15.700	0.85 ± 0.05[j]

Means within the same column followed by the different letter are significantly different ($p <$ 0.05) according to Duncan's multiple range test.

Figure S1. Heatmap generated with OrthoANI values calculated from the OAT sofware among *Streptomyces* sp. AN090126 with other type strain of Streptomyces species

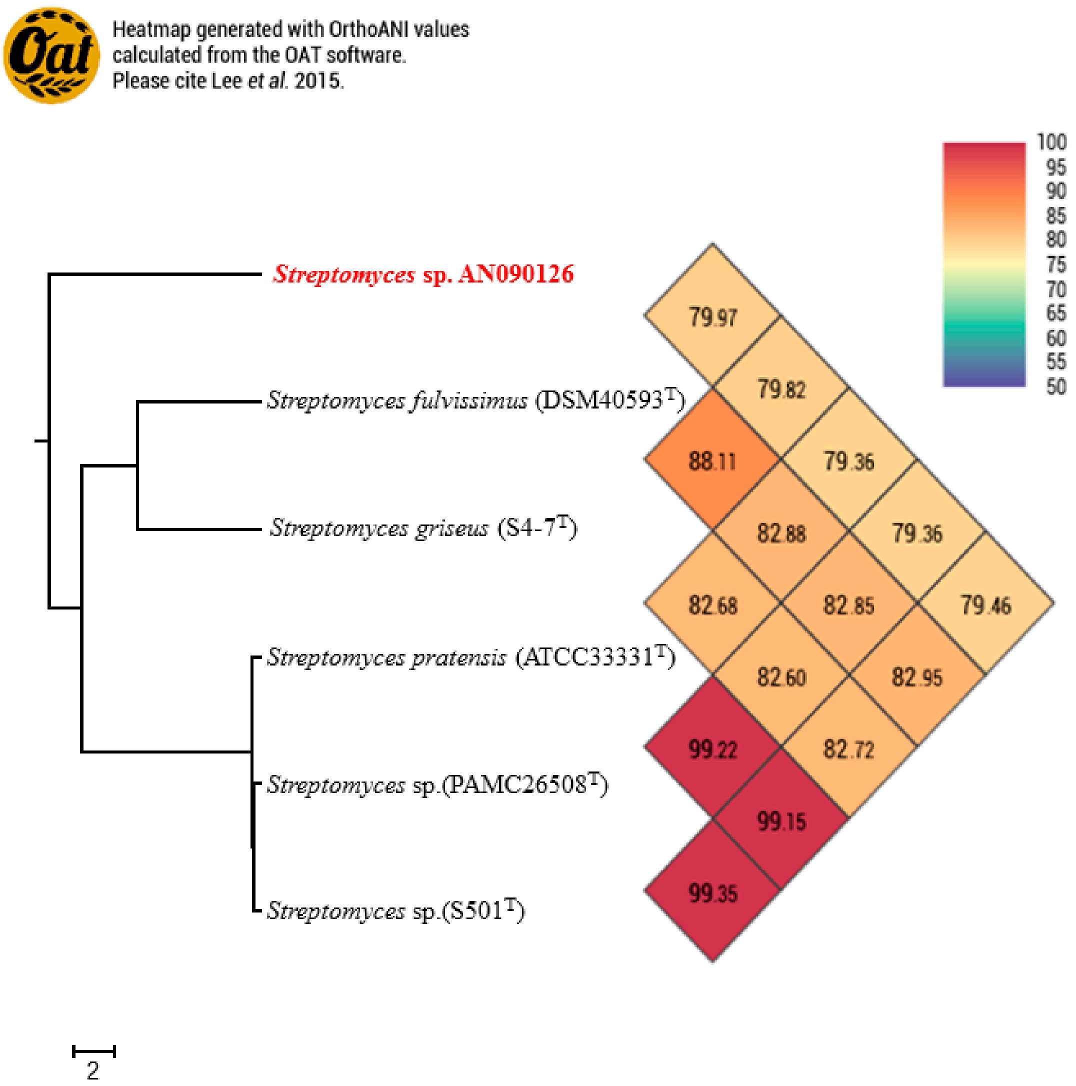

Figure S2. Results of total ion chromatography and gas chromatography–mass spectrometry showing differences in peaks between *Streptomyces* sp. AN090126 and control (ISP2 medium) (**A**). Microscopic observation of *Fusarium graminearum* hyphae after 4 days of exposure to volatile organic compounds produced by *Streptomyces* sp. AN090126 (**B**).

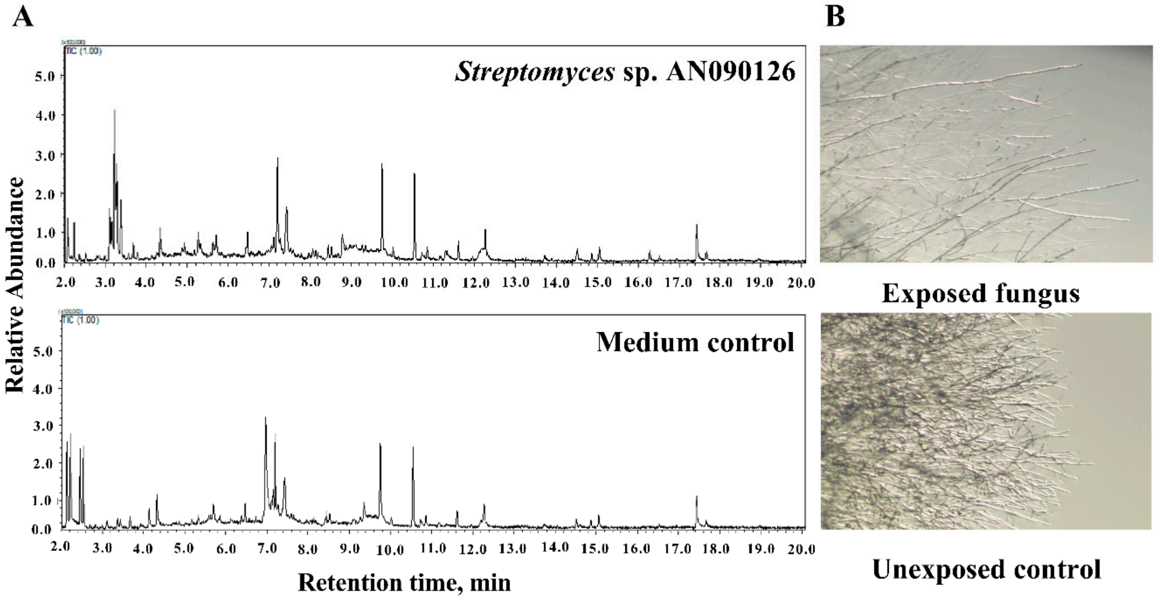

Figure S3. Effect of temperature on the antimicrobial activity of active substances (**A**). Bioautography of active substances on paper thin-layer chromatography using a butanol:acetic acid:water (4:1:5) solvent system (**B**). BA = antibacterial activity; FA = antifungal activity; *Acidovorax avenae* subsp. *cattleyae* and *Fusarium graminearum* were used as the indicators.

A **B**

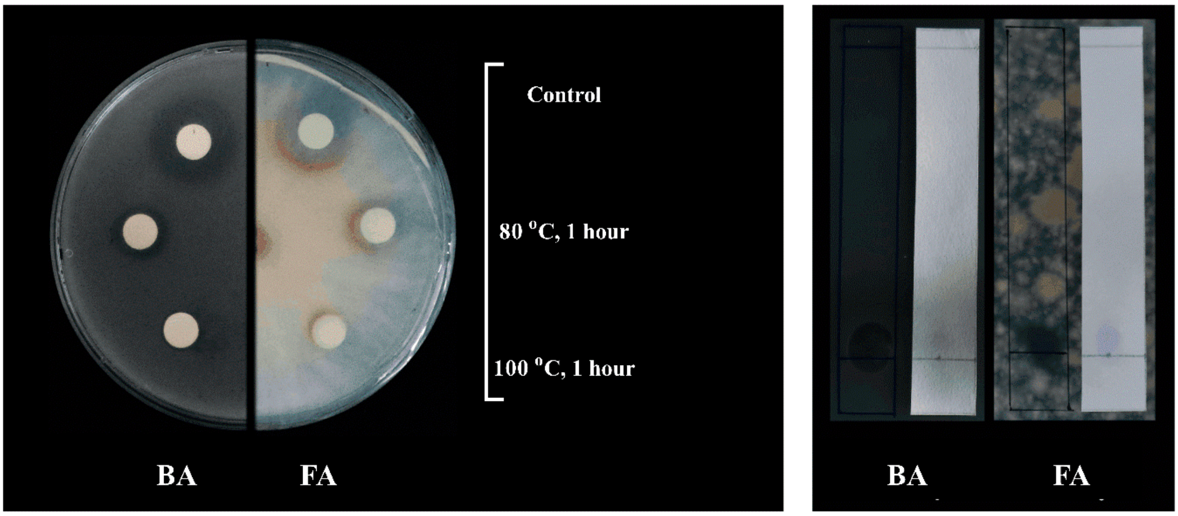

Supplement: Supplementary file 1 [file microorganisms-10-00791-s001.zip › microorganisms-1664522-supplementary.pdf]
